# Recurrent pyothorax in a cat caused by *Candida albicans*

**DOI:** 10.1177/20551169251383150

**Published:** 2025-12-02

**Authors:** Julie van Duijl, Chiara Valtolina, Alma Hulsman, Sanne Hugen

**Affiliations:** 1 Section of Emergency and Critical Care, Department of Clinical Sciences, Faculty of Veterinary Medicine, Utrecht University, The Netherlands; 2 Section of Cardiology, Department of Clinical Sciences, Faculty of Veterinary Medicine, Utrecht University, The Netherlands

**Keywords:** *Candida albicans*, pyothorax, *Candida* empyema, immunocompetence

## Abstract

**Case summary:**

A 7-month-old Siberian cat was presented for persistent fever and recurrent tachypnoea and dyspnoea. The cat was treated 2 weeks prior by the referring veterinarian for pyothorax. Repeated thoracic radiographs and CT showed severe pleuropneumonia. Although serology was positive for feline coronavirus antibodies, an RT-PCR for feline infectious peritonitis of the thoracic fluid was negative. The feline immunodeficiency virus/feline leukaemia virus SNAP test was negative. Repeated aerobic and anaerobic culture and sensitivity results were unable to detect bacterial growth but did detect *Candida albicans*. Initial treatment with amphotericin B and long-term treatment with itraconazole resulted in complete and persistent clinical recovery.

**Relevance and novel information:**

Pyothorax by *C albicans* in a cat has been described once in 1984. To the authors’ knowledge, this is only the second case description, confirmed by fungal culture.

## Introduction

Feline pyothorax is a respiratory emergency, diagnosed in approximately 10% of dyspnoeic cats^1^ and characterised by the accumulation of septic exudative fluid in the pleural cavity. The two most common aetiologies in cats include penetrating thoracic wounds, secondary to a cat scratch or bite, and parapneumonic spread of oropharyngeal bacteria in cats with chronic upper airway infection.^2^ Other suggested aetiologies are foreign body migration, parasitic infections (eg, *Aelurostrongylus abstrusus, Cuterebra* species, *Toxocara cati*), iatrogenic causes (eg, oesophageal feeding tube misplacement, postoperative or post-thoracocentesis infection) and, less frequently, haematogenous or lymphatic spread from systemic disease. Multi-cat households show an increased prevalence.^3–7^

Multiple anaerobic and aerobic bacteria (*Pasteurella* species, *Bacteroides* species, *Actinomyces* species, *Clostridium* species, *Fusobacterium* species, *Prevotella* species and *Peptostreptococcus* species) are often concurrently isolated in feline pyothorax, highlighting the importance of requesting both bacterial culture methods.^2,8,9^ *Candida albicans* has been reported in one single case report as the causative agent.^10^ *C albicans* is a budding yeast, part of the commensal flora of the oral, upper respiratory, gastrointestinal and genital mucosa of cats, dogs and humans.^11^ It can become pathogenic as a result of disruption of the host’s immune system, local defence mechanisms or expression of virulence factors.^11^ Fungal empyema is typically uncommon in both human and veterinary medicine.^10,12^

The following case report describes a cat with recurrent pyothorax due to *C albicans*.

## Case description

A 7-month-old male neutered indoor Siberian cat was presented to the emergency department of the Small Animal Hospital, University of Utrecht, the Netherlands, for persistent fever and recurrent tachypnoea.

Two weeks prior, the cat had been treated at a referral hospital for suspected bacterial pyothorax, as intracellular bacteria were observed on cytology. However, bac-teriology (blood agar) remained negative, but did reveal the presence of *C albicans*. No action was taken in response to this finding. At that time, initial diagnostics included complete blood count, serum biochemistry and protein electrophoresis, all of which showed no significant abnormalities. The feline immunodeficiency virus (FIV)/feline leukaemia virus (FeLV) SNAP test (SNAP* combo plus; IDEXX) was negative. Treatment included the placement of bilateral chest drains, lavage and a combination of cefazolin (25 mg/kg IV q12h, Kefzol; Eurocept), metronidazole (15 mg/kg IV q12h, Metrobactin; Dechra) and meloxicam (0.05 mg/kg IV q24h, Metacam; Boehringer Ingelheim) for 8 days. Despite clinical improvement, the fever persisted. Follow-up thoracic radiographs showed a reduction of the pleural effusion. Feline coronavirus serology was positive, although RT-PCR for feline infectious peritonitis (FIP) on the pleural fluid was negative. Chest drains were removed, and the cat was discharged with metronidazole (same dose) for 1 day, amoxicillin/clavulanic acid (12.5 mg/kg PO q12h, Synulox; Zoetis) for 4 days and meloxicam (same dose) for 5 days. On the owner’s initiative, daily subcutaneous GS-441524 injections (unregistered formulation) were administered for 3 days. However, recurrent tachypnoea, dyspnoea and lethargy prompted referral to Utrecht University.

Upon arrival, the cat appeared bright and alert but had mild tachypnoea with expiratory dyspnoea. Auscultation revealed mildly reduced lung sounds cranioventrally. Cardiovascular stability was noted, with a normal rectal temperature. Thoracic point-of-care ultrasound (POCUS) revealed a small amount of pleural fluid and coalescing B-lines in the right hemithorax. Thoracocentesis removed 13 ml of purulent fluid. Cytology (May–Grünwald–Giemsa) confirmed a neutrophilic inflammation. Echocardiography, performed by a board-certified cardiologist, ruled out cardiac disease. Radiographs showed mild bilateral pleural effusion (R > L) with a generalised bronchointerstitial pattern. The owners declined additional advanced diagnostics and hospitalisation at this point and opted for continued medical management. The therapy with amoxicillin/clavulanic acid and meloxicam was continued.

Two days later, the cat was readmitted with marked expiratory dyspnoea, tachypnoea and fever (40.2°C). Auscultation revealed increased bronchovesicular lung sounds bilaterally. No cardiovascular abnormalities were noted. The cat was admitted to the intensive care unit and placed in an oxygen cage. Blood work revealed a mild non-regenerative, microcytic, hypochromic anaemia (haematocrit 26%), suggestive of iron deficiency anaemia, while plasma biochemistry results were unremarkable. Repeated radiographs showed a diffuse alveolar pattern, obscuring the cardiac silhouette and diaphragm and pleural fissure lines, suggestive of bronchopneumonia with pleural effusion (R > L). Ultrasound-guided thoracocentesis removed some additional fluid. Cytology (May–Grünwald–Giemsa) was negative for organisms. The sample was submitted for bacteriology (blood agar) and fungal culture (Sabouraud dextrose agar). Initial treatment included intravenous fluid therapy, amoxicillin/clavulanic acid (20 mg/kg IV q8h, Amoxicilline/Clavulaanzuur; Sandoz) and continued meloxicam (0.05 mg/kg PO q24h, Metacam; Boehringer Ingelheim). Daily POCUS showed persistent, although reduced, pleural effusion localised to the right hemithorax. However, further diagnostics and no improvement prompted the placement of bilateral chest drains (guidewire inserted chest tube 10 Ga; MILA International) under general anaesthesia.

**Figure 1:**
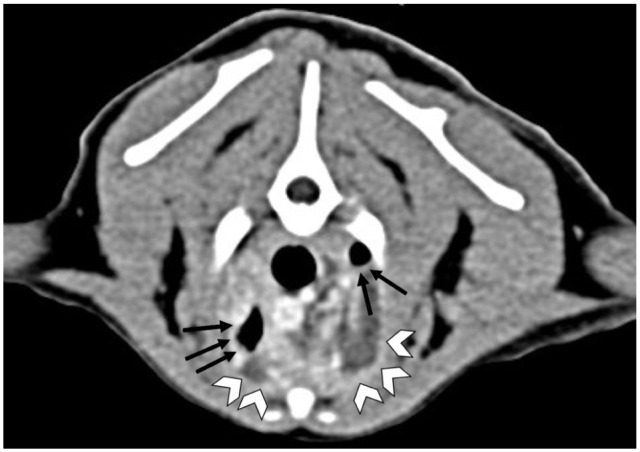
Thoracic transverse reconstruction of a post-contrast soft tissue algorithm at the level of the third rib. Fluid attenuating material with peripheral contrast enhancement (arrowheads) is observed in the ventral aspect of the pleural space (left > right), causing retraction of the lung parenchyma (arrows)

**Figure 2:**
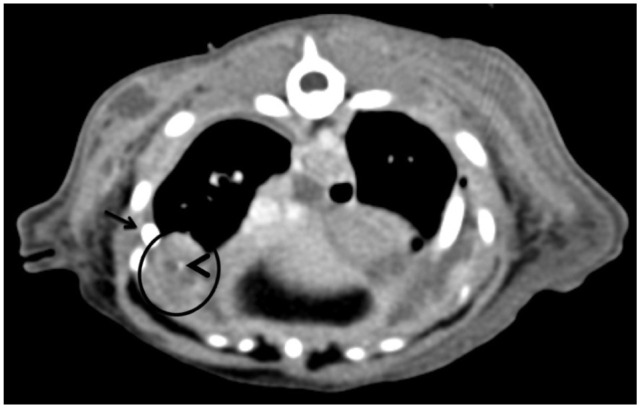
Thoracic transverse reconstruction of a post-contrast soft tissue algorithm at the level of the 10th rib. A small mineralisation is observed in the ventral aspect of the right caudal lung lobe (arrowhead), within a well-defined fluid structure with peripheral contrast enhancement (circle). Part of the pleural catheter is observed (arrow), as well as a mild volume of pleural effusion

Thoracic CT (Figures 1 and 2) imaging revealed an alveolar pattern in the right accessory and left cranial lung lobes, moderate pleural effusion and nodular mineralised changes in the ventral portions of the right middle and caudal lung lobes, consistent with severe pleuropneumonia.

A foreign body was considered less likely. Bacterial culture remained negative, but *C albicans* was confirmed via fungal culture. Amphotericin B (0.25 mg/kg IV q48h, Fungizone; Cheplapharm) was initiated. Renal parameters were monitored daily because of amphotericin B’s nephrotoxicity, and a mild increase in creatinine levels (from 68 to 102 μmol/l, reference interval [RI] 76–164) was noted. Urinalysis revealed mild proteinuria (0.5, RI < 0.4), granular casts (0–1 per high power field) and epithelial cells, consistent with acute kidney injury (AKI) grade I with proteinuria. Fluid balance was monitored and renal parameters stabilised. Meloxicam was stopped and switched to buprenorphine (10 µg/kg IV q6h, Bupaq; Vetviva Richter). Daily chest lavage reduced pleural effusion, and after 5 days, chest drains were removed. The cat remained stable without supplemental oxygen therapy. One day later, recurrent fever (39.5°C) was noted and two subcutaneous masses were palpated at prior chest drain sites. An ultrasound-guided aspiration of a subcutaneous fluid pocket was performed and cytology showed a non-septic exudate. Bacterial culture (blood agar) showed an overgrowth of *C albicans*. Although there was intermittent fever and the subcutaneous masses remained similar in size, shape and consistency, the cat was eating and drinking and showed no respiratory signs. He was discharged with itraconazol (5 mg/kg PO q24h, Itrafungol; Janssen Pharmaceutica) for 7 weeks and amoxicillin/clavulanic acid (12.5 mg/kg PO q12h, Synulox; Zoetis) for 5 days.

**Figure 3:**
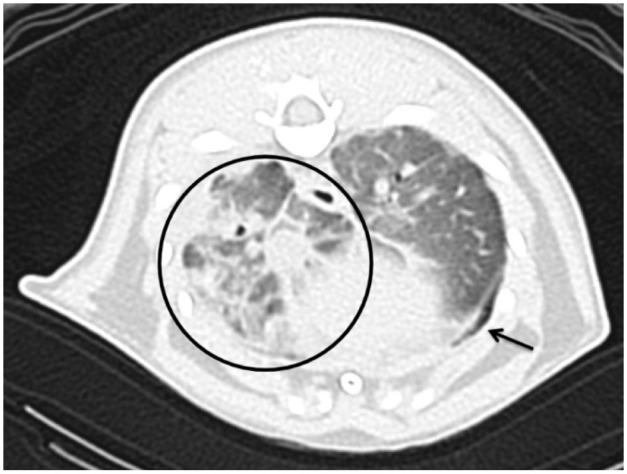
Thoracic transverse reconstruction in lung algorithm at the level of the caudal lung lobes. Pleural thickening is observed (arrow), as well as a patchy and peribronchial alveolar pattern (circle)

At a 6-week recheck, the cat displayed daily coughing but no respiratory distress. Physical examination revealed no abnormalities. Blood work, including kidney and liver parameters, remained normal. CT imaging (Figure 3) revealed minimal pleural effusion, improvement in the alveolar pattern of the cranial lung lobes and worsening of patchy changes in the right caudal lobe. Mineralised nodules remained unchanged. Itraconazole was continued.

Five months after treatment, coughing had resolved, but mild inspiratory stridor and sneezing were noted. Physical examination revealed slight tachypnoea but normal lung sounds. Radiographs showed a mild bronchointerstitial pattern, suggesting continued improvement. Itraconazole was discontinued after 1 more month of treatment.

**Figure 4:**
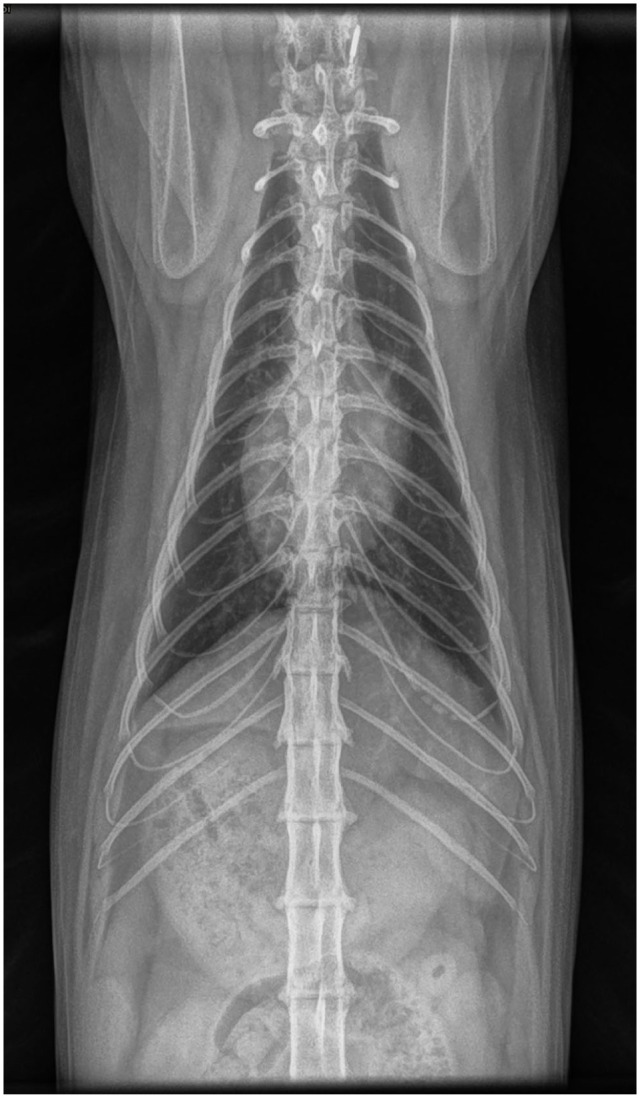
Dorsosagittal view of the thorax. Lung parenchyma is well-aerated and expanded. The pleural space is unremarkable

**Figure 5:**
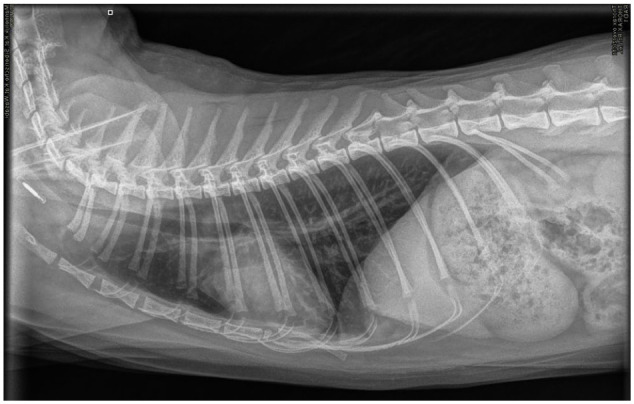
Dorsoventral view of the thorax. Lung parenchyma is well-aerated and expanded. The pleural space is unremarkable

At a 3-year recheck, the cat was alert and had gained weight. Only an inspiratory stertor was noted, likely due to chronic rhinitis. Radiographs (Figures 4 and 5) revealed normal lung parenchyma with an unremarkable pleural space.

## Discussion

This case report describes a 7-month old Siberian cat with *Candida* empyema and is, as far as the authors are aware, the second one published since 1984.

As mentioned earlier, *Candida* species are commensal fungi that need specific conditions to become pathogenic. Although immunosuppression has traditionally been considered a prerequisite for infection, previous studies on feline candidiasis have reported cases in immunocompetent cats, as seen in this case.^10,13–15^ The cat tested negative for FIV, FeLV and FIP, and no indications of systemic immunosuppression, infectious diseases or endocrinopathies (such as diabetes mellitus or hypercortisolism) were identified. In addition, the cat had not received medications that could suppress his immune system. However, an underlying genetic immunodeficiency could not be ruled out.^11^

Diagnosis in this case was made based on persistent dyspnoea, fever and pleural effusion, despite antibiotic therapy, and growth of *C albicans* on bacteriology on two separate occasions, confirmed by fungal culture. This corresponds closely with earlier criteria for the diagnosis of fungal empyema in humans.^12^ Although cytology has been instrumental in diagnosis in earlier reports,^12^ it failed to detect *C albicans* in our case. The use of fluorescent brightener staining has been shown to improve diagnostic sensitivity^16^ and might have enabled a more rapid diagnosis and initiation of treatment. However, this method was not available in our laboratory at the time.

Although blood cultures were not performed, in a retro-spective study of human medicine they are rarely *Candida*-positive. This suggests that *Candida* empyema is not necessarily related to candidemia and that blood culture is not a substitute for fungal culture of pleural fluid. Histopathology of lung tissue was also not conducted because of financial constraints, although it is not a necessary criterion for diagnosing *Candida* empyema, as the condition is rarely a consequence of pneumonia.^17^

In human medicine, first-line antifungal treatment for invasive candidiasis typically involves echinocandins (beta-D-glucan synthase inhibitors), although their pene-tration in pleural fluid is poor.^16,18^ Higher pleural concentrations were achieved with liposomal amphotericin B and voriconazole.^18^ However, an optimal treatment protocol for *Candida* empyema has not yet been established. In a study of human patients, Senger et al^16^ were the first to identify a preferred antifungal agent, reporting better outcomes in those treated with fluconazole.

We opted for amphotericin B as the initial therapy, followed by multiple months of itraconazole. The choice for amphotericin B was made based on availability, cost, route of administration (intravenous instead of per os for itraconazole) and stability after preparation. Despite the clear clinical improvement, we did see an initial rise in creatinine and urea, consistent with AKI grade I, highlighting the nephrotoxicity of amphotericin B and the importance of monitoring the renal parameters during treatment.^19^

Fluconazole is the preferred antifungal for cats because of its safety profile in comparison with other azoles.^19^ Furthermore, its penetration into pulmonary epithelial lining fluid in cats is high.^20^ Voriconazole is not advised for use in cats.^19^ The other azoles have a particularly high protein binding rate, which could limit their distribution into lung tissue, although their lipophilicity is of benefit.^19,21^ There are no other studies about pleural penetration of antifungal drugs in cats; therefore, based on availability, we opted for itraconazole.

Treatment duration was grounded on clinical signs and radiographic findings, with a significantly longer treatment period (6 months) compared with the previously reported case (35 days). This discrepancy may be due to a longer follow-up and more intensive monitoring, although the absence of repeat fungal cultures leaves some uncertainty about whether the prolonged therapy was fully justified. In human medicine, candidemia treatment is typically continued for 14 days after negative blood culture and resolution of clinical signs, with a mean therapy duration of 21 days for *Candida* empyema.^16,17^

## Conclusions

*Candida* empyema is a rare disease in both humans and cats, with only a few published articles to date. Clinical signs of persistent fever despite antibiotic treatment should warrant further diagnostics for the clinician. Consideration should be given to using special stains on cytology in cases with an atypical presentation. Diagnosis should be confirmed by fungal culture. In cats with recurrent/unresolved pyothorax, *C albicans* is a differential that should be considered.
